# A decade of outcomes: The evolution of an australasian outcomes collaboration for chronic pain services

**DOI:** 10.3389/fpain.2023.1153001

**Published:** 2023-04-12

**Authors:** David Holloway, Samuel Allingham, Meredith Bryce, Kate Cameron, Michelle Cook, Dinberu Shebeshi

**Affiliations:** Electronic Persistent Pain Outcomes Collaboration, Australian Health Services Research Institute, University of Wollongong, Wollongong, NSW, Australia

**Keywords:** quality improvement, outcome assessment, chronic pain, benchmarking, quality improvement outcomes

## Abstract

Since the establishment of the electronic Persistent Pain Outcomes Collaboration (ePPOC) in 2013, ongoing improvements in benchmarking and quality improvement activities have provided the opportunity for ePPOC to grow to support more than one hundred adult and pediatric services delivering care to Individuals living with persistent pain throughout Australia and New Zealand. These improvements straddle multiple domains, including benchmarking and indicators reports, internal and external research collaboration and the integration of quality improvement initiatives with pain services. This paper outlines improvements undertaken and lessons learned in relation to the growth and maintenance of a comprehensive outcomes registry and its articulation with pain services and the wider pain sector.

## 1. Introduction

The electronic Persistent Pain Outcomes Collaboration (ePPOC) was formed in 2013 to ensure benchmarking and outcomes were part of a growing agenda to improve the quality of life for people living with persistent pain in Australia (AU) and New Zealand (NZ) (1). The Faculty of Pain Medicine of the Australian & New Zealand College of Anaesthetists identified the need for a national outcomes data set to progress benchmarking and quality improvement. On development of an Australian National Pain Strategy (NPS), there were clear objectives on quality improvement through outcomes measurement and benchmarking and acknowledgement that this needed to occur in both the adult and paediatric populations. The achievement of this objective was realised in 2012, with the NSW Agency for Clinical Innovation Pain Management Network (part of the New South Wales Ministry of Health) funding the simultaneous implementation of both an adult dataset (ePPOC) and pediatric dataset (PaedePPOC) (2).

In the decade since the establishment of ePPOC and PaedePPOC, there have been ongoing improvements made in respect of data items captured and the processes supporting the submission of data and reporting of outcomes. These systemic improvements, combined with the active engagement of member services, allows for transparent discussions on individual service outcomes and the identification of quality improvement initiatives to bolster outcomes further. This paper will outline the core components of ePPOC, the use of patient-reported outcomes data for clinical and quality improvement, and the research outputs achieved.

## 2. Data collection approach

### 2.1. Assessment and feedback

ePPOC collects a standard set of information by specialist pain management services from across AU and NZ. This information is used to guide treatment for individual patients, measure outcomes following treatment and provide a benchmarking system across nine domains. The benchmarking system is designed to provide comparative outcomes and service process data to each pain service, identify best practice protocols and clinical variation, and drive quality improvement through setting aspirational targets for patient and service outcomes. ePPOC's ethics approval allows for the collection of patient de-identified outcome and specialist pain service data from participating services, for the purposes of reporting, quality assurance, and benchmarking. Specialist pain management services also seek advice from their relevant human research ethics committee where relevant (1).

**Figure 1:**
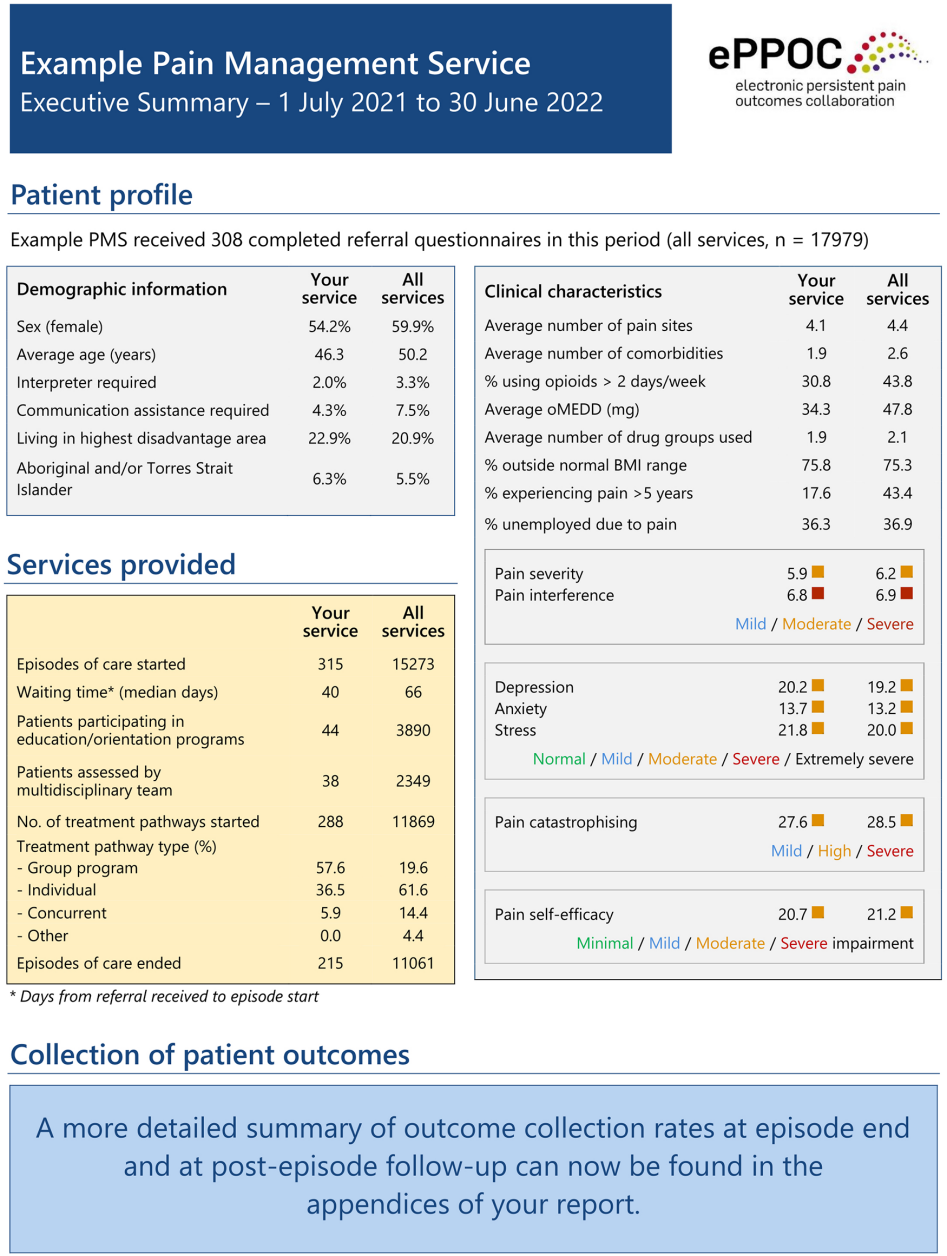
Example of one part of standardized report.

Participating services receive twice-yearly standardized reports and stand-alone executive summaries and dashboards, summarizing their achievement against outcome measures with established benchmarks. The reports also allows services to anonymously compare their results against other participating services (see Figure 1 for an example of an executive summary). On receipt of each report, pain services can also access support from ePPOC's Improvement Facilitators (IFs). As will be discussed below, IFs work with services to analyze their results, identify areas of high performance, areas in need of improvement and relevant quality projects and strategies.

### 2.2. The ePPOC and PaedePPOC data collections

Central to ePPOC's cycle of assessment and feedback is the ePPOC data collection, which was designed to capture clinically meaningful information, with an emphasis on patient reported outcome measures (PROMs) in both the adult and pediatric programs, with additional carer-proxy outcomes measures in the pediatric program. The PROMs and carer proxy measures provide an assessment of the health status of a patient at a prescribed time during their treatment, with paired information pre- and post- treatment used to measure and benchmark patient outcomes.

Patients also provide information relating to the cause of their pain, how long it has been a problem, and where and how widespread it is (*via* a pain body map). Medication use and health service utilization in the three months prior to completing the referral questionnaire is also captured, as is information about the impact of pain on work productivity and employment. The pediatric data set includes carer reported data on the impact of the child's pain on school and carer work productivity and employment. Patient (and carer) reported information is supplemented with clinician reporting of the type and duration of treatments the patients received *via* treatment pathways and service events.

Table 1 summarizes the characteristics of adult patients (age greater than 18 years). The patient's work and productivity status showed that the majority of patients were not working due to pain at referral. The most commonly reported pain site was the back. Mental health condition was reported as the main comorbidity for chronic pain patients. Other commonly reported comorbidities included arthritis, “heart problems”, “digestive problems”, “respiratory problems” and high blood pressure.

**Table 1:** Characteristics of adult patients included in the analysis (2014–2021).

Characteristics of patients	Australia (*N* = 109,013)	New Zealand (*N* = 32,546)	Total (*N* = 141,559)
Gender, females, *N* (%)	63,956 (58.8)	17,721 (54.6)	81,677 (57.8)
**Age in years, mean (SD)**			
- Male	51.6 (14.9)	45.5 (13.5)	50.1 (14.8)
- Female	52.4 (16.1)	45.1 (14.6)	50.8 (16.1)
Body Mass Index, mean (SD)	30.1 (11.1)	30.4 (15.7)	30.1 (12.3)
**Country of birth, *N* (%)**			
- Australia, New Zealand^a^	75,397 (70.6)	24,722 (77.7)	103,313 (74.5)
- Other	31,409 (29.4)	31,409 (22.3)	35,293 (25.5)
Aboriginal or Torres Strait Islander, *N* (%)	4,659 (4.5)	-	4,659 (4.5)
Maori, N(%)	**-**	4,394 (13.5)	4,394 (13.5)
**Work status, *N* (%)^b^**			
- Not working due to pain	37,027 (36.7)	12,347 (41.0)	49,374 (37.7)
- Working full-time	12,891 (12.8)	7,395 (24.5)	20,286 (15.5)
- Working part-time	12,329 (12.2)	4,862 (16.1)	17,191 (13.1)
- Not working due to a condition other than pain	18,023 (17.9)	2,760 (9.2)	20,783 (15.9)
- Not working by choice/seeking employment	20,695 (20.5)	2,769 (9.2)	23,464 (17.9)
**Main pain site, *N* (%)**			
- Back	39,270 (44.5)	10,018 (34.6)	49,288 (42.1)
- Abdomen	9,804 (11.1)	3,056 (10.6)	12,860 (11.0)
- Leg	4,450 (5.0)	2,612 (9.0)	7,062 (6.0)
- Neck	7,290 (8.3)	1,486 (5.1)	8,776 (7.5)
- Arm/shoulder	1,457 (1.7)	1,367 (4.7)	2,824 (2.4)
- Head	4,601 (5.2)	1,088 (3.8)	5,689 (4.9)
- Other	21,364 (24.2)	9,293 (32.1)	30,657 (26.2)
**Cause of pain (precipitating event), *N* (%)**			
- Injury	37,513 (35.4)	20,866 (66.0)	58,379 (42.5)
- Motor vehicle accident	11,230 (10.6)	2,585 (8.2)	13,815 (10.0)
- After surgery	9,306 (8.8)	2,476 (7.8)	11,782 (8.6)
- No obvious cause	18,577 (17.5)	1,860 (5.9)	20,437 (14.9)
- Medical condition other than cancer	14,562 (13.7)	1,849 (5.9)	16,411 (11.9)
- Cancer	1,720 (1.6)	147 (0.5)	1,867 (1.4)
- Other	13,006 (12.3)	1,813 (5.7)	14,819 (10.8)
Patients experiencing pain more than 5 years, *N* (%)	50,267 (47.6)	7,634 (24.2)	57,901 (42.2)
**Comorbidities, *N* (%)^b^**			
- Mental Health condition	52,138 (47.8)	8,861 (27.2)	60,999 (43.1)
- Arthritis	41,402 (38.0)	4,599 (14.1)	46,001 (32.5)
- Heart and circulation problems	30,426 (27.9)	3,971 (12.2)	34,397 (24.3)
- Digestive problems	24,401 (22.4)	4,448 (13.7)	29,022 (20.5)
- Respiratory problems	21,378 (19.6)	4,448 (13.7)	25,826 (18.2)
- High blood pressure	20,920 (19.2)	2,524 (7.8)	23,444 (16.6)
- Diabetes	11,914 (10.9)	1,759 (5.4)	13,673 (9.7)
- Neurological problems	8,662 (7.9)	1,305 (4.0)	9,967 (7.0)
- Liver, kidney and pancreas	7,439 (6.8)	998 (3.1)	8,437 (6.0)
- Cancer	5,088 (4.7)	559 (1.7)	5,647 (4.0)
- Other medical problems	19,908 (18.3)	4,328 (13.3)	24,236 (17.1)

^a^ Country of birth percentage presented separately for Australia and New Zealand;.

^b^ Note: will not add to 100% as multiple categories may be chosen.

Information collected in both the adult and pediatric data collections have been reported previously (1, 2), with a summary of the patient and carer-reported outcome measures used in both the adult and pediatric data collections provided in Table 2. Each measure is captured at referral, episode end and 3–6 months after completion of treatment where feasible.

**Table 2:** Patient/carer reported measures for the adult and paediatric data collections.

Domain	Adult data collection	Paediatric data collection	
	Patient reported	Patient reported	Parent/carer reported
Pain severity	Brief Pain Inventory—Pain Severity Items	Modified Brief Pain Inventory (patients aged 8-18yo)	Modified Brief Pain Inventory (all ages)
		Faces of Pain Scale-R (patients aged 5-7yo)	
Pain interference	Brief Pain Inventory—Interference Items	-	-
Pain catastrophising	Pain Catastrophising Scale (PCS)	-	-
Pain self-efficacy	Pain Self Efficacy Questionnaire (PSEQ)	-	-
Pain location	CARRA Body Map	CARRA Body Map	CARRA Body Map
Health Related Quality of Life (HRQOL)	-	PedsQL	PedsQL
Emotional distress	Depression, Anxiety Stress Scale (DASS)	-	Bath Adolescent Pain Questionnaire—Pain Related Worries
Patient's Impression of Change due to treatment	ePPOC Patient Impression of Change (ePIC) tool	-	-
Physical functioning and disability	-	Functional Disability Inventory (FDI) (patients aged 8-18yo)	-
Disabilities	-		
Comorbidities			
Medication usage	Indicator of usage for key drug groups^a^	-	Frequency of use for key drug groups
	Opioid frequency^a^		
	oral Morphine Equivalent Daily Dose (in mg)^a^		
Healthcare utilisation	Utilization of specified health care services in the last 3 months	-	Utilization of specified health care services in the last 3 months
Impact of pain on school	-	-	School day missed in previous fortnight
Impact of pain on work	Work Productivity and Activity Impairment (WPAI)	Work Productivity and Activity Impairment (WPAI)	Work Productivity and Activity Impairment (WPAI)
Impact of pain on parents/carers	-	-	Bath Adolescent Pain—Parental Impact Questionnaire (BAP-PIQ)

^a^ derived by clinician/staff member from patient reported medication usage.

**Figure 2:**
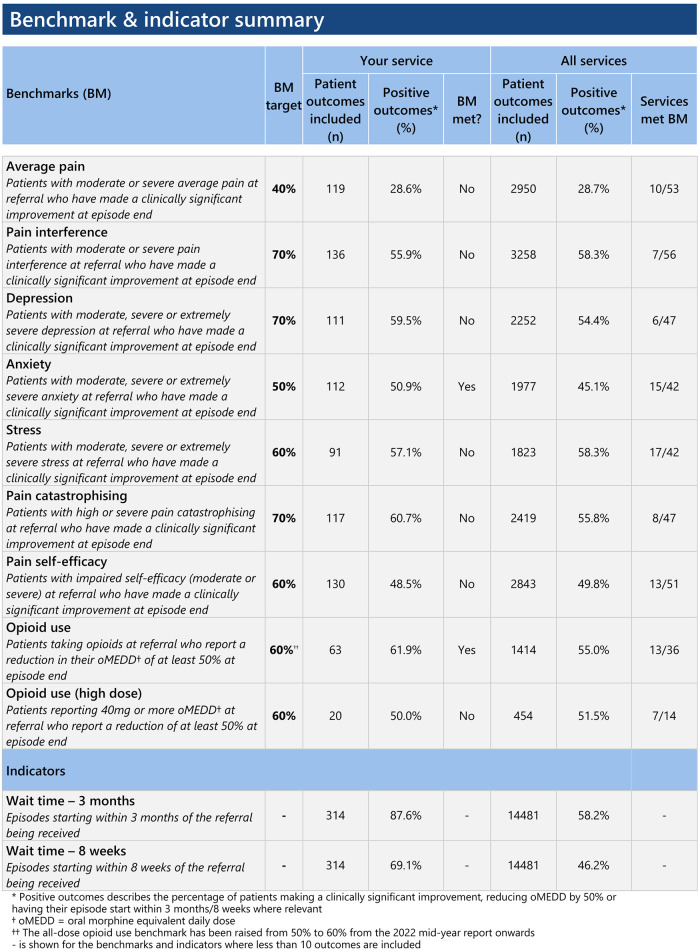
Clinical outcomes by domain, total patient cohort 2021.

An example of clinical outcomes across the full patient cohort is provided in Figure 2, where the level of improvement for pain interference, depression, anxiety and stress from referral to episode end is illustrated for the total adult cohort at end of 2021 reporting period.

### 2.3. PROMs included in the adult data collection

The PROMs collected in the adult data collection align with the core domains recommended by the Initiative on Methods, Measurement, and Pain Assessment in Clinical Trials (IMMPACT) (3) and have been reported in detail previously (1). These domains and measures are:

Pain, measured by the pain severity elements of the Brief Pain Inventory (BPI) (4).
Physical functioning, measured by the BPI Interference items (4).
Emotional functioning, as measured by the Depression, Anxiety and Stress Scale (DASS) (5) and the Pain Catastrophizing Scale (PCS) (6).
The Pain Self-Efficacy Questionnaire (PSEQ) (7) is also included, to provide an assessment of how confident patients feel in performing activities despite their pain.

These outcomes are complimented by the ePPOC Patient Impression of Change (ePIC) assessment tool, which provides patients who have received treatment an opportunity to reflect on their perception of change, both overall and in respect to physical abilities.

### 2.4. PROMs and carer proxy measures: pediatric data collection

The PROMs and carer proxy measures included in the pediatric data collection were informed by the domains outlined in the PedIMMPACT Consensus statement (8). However, the pediatric data collection is more complicated than for adults, with patient and carer-reported tools catering to different age groups and used in combination in some cases. The pediatric tools cover the domains of pain severity, pain related anxiety, functional disability, and health related quality of life (physical, social, emotional and school functioning). The development of the current version of the data collection (version 2), including the rationales for tool selection has been previously documented (2).

### 2.5. Timing of assessment

The adult and pediatric data collections follow the same assessment protocol, with the patient (and carer) questionnaires administered at referral to the pain service and then at multiple time points once treatment has commenced, including the start and end of treatment pathways and the end of the overall episode of care. A follow-up questionnaire is administered 3–6 months after the episode has ended, to ascertain whether improvements from treatment have been maintained. Clinicians can also administer “clinical review” questionnaires at any time during treatment, if it is felt the additional information would be useful in planning care, or if a patient experiences a significant change in their condition.

The pediatric data collection also includes an assessment of the impact on the parent/carer of their child's pain. The timing of parent/carer-impact data collection varies slightly, with information collected at referral, 12-months following pathway start, pathway end, at the end of treatment and 3–6 months after the episode has ended.

### 2.6. Measuring patient outcomes

Patient outcomes are measured using a pre-/post- methodology, with the benchmarked patient outcome measures focused on the changes between referral to the pain service (pre-treatment) and at end of the episode of care (post-treatment). A patient is deemed to have achieved a positive outcome on a measure if they report a clinically significant improvement following treatment, or have reduced their average daily opioid intake by 50% or more. What constitutes a clinically significant improvement has been defined separately for each of the measures. For example, in relation to pain interference, a change of one point or more over the average of the seven interference items points to clinically significant change. These definitions are provided in a clinical reference manual provided to services and is also available publicly online (9).

ePPOC PROMS provides useful information to clinicians to inform care for a patient. The clinical utility of the data collection anecdotally increases both service staff and patient engagement, in turn leading to higher quality data provision by services as the value of the data is illustrated.

### 2.7. Epicentre computer application

As reported previously (1) ePPOC developed a software application called epiCentre (the ePPOC Patient Information Centre) which comprises a desktop application paired with a cloud-based REDCap server, used to deliver online questionnaires to patients/carers. In addition to supporting routine patient outcome reporting, epiCentre provides clinicians with the ability to generate progress reports for individual patients and to track improvement over time, with examples of all questionnaires provided by ePPOC online for researchers interested in examples (9). These individual-level reports are designed to enhance patient engagement with the pain service and support discussions between patients and their treatment providers about the care they are receiving and the progress they are making. Over the ten years of ePPOC's existence, the epiCentre application has been iteratively improved as feedback is received from services, further improving both quality of data and time for submission of data.

## 3. Australian and New Zealand pain service profile

**Figure 3:**
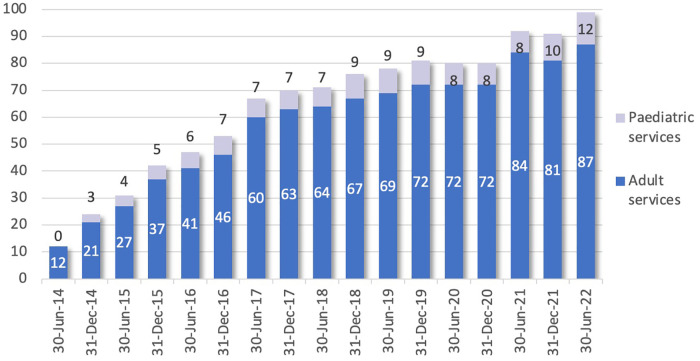
Number of data submitting services since ePPOC establishment.

Since establishment in 2013, there has been significant growth in the number of participating services. Pain management services who hold membership with ePPOC are dispersed across most states and territories of AU and throughout NZ. Figure 3 shows the accumulation of data-submitting services since ePPOC establishment in 2013. There are a small number of non-data submitting member services, which takes the total service membership above 100. The number of adult services increased from 21 in 2014 to 87 in 2022, while pediatric services increased from 3 in 2014 to 12 in 2021.

ePPOC's dataset now includes 109625 patient-completed questionnaires collected by Australian adult pain services at time of referral, with a further 32,923 adult questionnaires collected in NZ. 2,920 questionnaires have been collected from AU pediatric patients and 65 from NZ (Table 1). Geographically, the majority of adult services are located in New South Wales (NSW) and Victoria, AU's two most populous states (10). Accordingly, 44,317 patients from NSW and 34,380 patients from Victoria services provided data at referral during the years 2014–2022. Most pediatric pain management services were located in NSW and Queensland, with only one NZ pediatric service. Table 3 outlines the number of services contributing patient data, by year.

**Table 3:** Services location and received referral questionnaires since ePPOC establishment (January 2014–June 2022).

	Adult Services			Paediatrics services
	No. of Services	Referral questionnaire	No. of Services	Referral questionnaire
**Australia**				
New South Wales	24	44,317	3	1461
Queensland	9	17,263	6	509
South Australia	5	3863	1	215
Tasmania	1	574	-	-
Victoria	23	34,380	2	732
Western Australia	8	9228	1	3
**New Zealand**	27	32,923	1	65

## 4. ePPOC governance and risk management

The involvement of key stakeholders in chronic pain management in the Australian and New Zealand context has been integral to the governance of ePPOC since establishment (1, 2). The format and structure of what is now known as the ePPOC Clinical Management Advisory Committee (CMAC) has evolved over the decade of ePPOC's operation. This evolution occurred as ePPOC's operations matured and grew—the initial national reference group overseeing the establishment of ePPOC divided into management advisory and clinical advisory groups, before being combined in 2018 into the CMAC in operation today (1). These changes reflected the need to adjust the scope of governance as ePPOC grew in size and robust processes were put in place for governance-related issues such as access to ePPOC data for research.

CMAC meets up to four times per year and incorporates key stakeholders from peak bodies, consumers and funders across Australia and New Zealand. CMAC provides approvals in relation to proposed research utilizing ePPOC data, provides input on strategic direction and is a valuable conduit more broadly in relation to chronic pain services and their own evolution. In respect to research, researchers provide a proposal requesting data access, which is initially reviewed by ePPOC staff and then submitted to CMAC for approval.

There remain significant risk factors for ePPOC, particularly in relation to recurrent funding. Current funders come from a range of state and government entities in Australia and New Zealand, with all funding on a fixed term basis. Each funder receives their own report with aggregated information on the performance of their funded service cohort—the degree of identifiability of services to funders is dependent on specific funding arrangements and policies of the funder. There are clear CMAC terms of reference outlining the need for disclosure of potential conflicts of interest and documentation of these in meeting minutes.

The short-term nature of funding arrangements creates challenges in respect to long-term planning and investment in resources for more substantial development of the ePPOC program. The current staffing numbers 6.4 full-time equivalent staff only—this creates its own challenges with resources to evolve the ePPOC program whilst maintaining a burgeoning membership and providing extensive reporting of data to each member service, their funders and to external researchers. Continuity of service provisions across the past ten years has been feasible due to growth in services participating and fortuitous timing of funding contracts.

## 5. Approach to quality improvement

### 5.1. Improvement facilitator role

As volume and diversity of chronic pain service membership grew, the need was identified for clinically trained staff who could work directly with member services to embed ePPOC protocols into existing clinical operations, provide ongoing support to ensure consistent high-quality practices, and facilitate use of the program to achieve continuous improvement. To address this, the role of Improvement Facilitator (IF) was created, and the first IF commenced in 2015.

The IF role requires health professionals with experience working in interdisciplinary, complex health settings. This experience affords knowledge of disciplines, interdisciplinary care, pain service operations, general health practices. In addition to an understanding of health care, IFs are also required to possess a firm understanding of continuous improvement principles and have intermediate competencies in data analysis and interpretation. Key Australian frameworks that guide ePPOC's approach to quality improvement are the National Safety and Quality Health Service (NSQHS) Standards (11) and the The National Strategic Action Plan for Pain Management (12). The IFs serve three key purposes: supporting services to embed ePPOC into their care delivery, providing ongoing support to services once they have embedded the approach, and finally acting as a conduit between the pain service and the broader ePPOC team. Each of these functions are described further below.

### 5.2. Embedding ePPOC into care delivery

When a new pain service joins ePPOC, the IF is responsible for introducing the principles and practices of ePPOC and aiding the integration of the epiCentre application within the pain service. The guidance required to effectively embed a new system into a dynamic clinic environment can be significant and includes training (face-to-face or virtual) on the key milestones for data collection, effective use of the epiCentre application, troubleshooting of any issues and liaison with the ePPOC team for resolution of those issues if required.

The initial training provided to a pain service is a useful quality improvement initiative in itself, providing the service an opportunity to review the patient journey and identify where there might be delays or gaps in progressing a patient from referral to commencement of treatment. Building rapport with all staff *via* meetings and other communication ensures a team understanding and engagement with ePPOC processes. Participation in ePPOC does involve extra administrative work for pain services, therefore reinforcing the benefits of robust data submission is an additional focus. A “whole team approach” ensures that data is collected at standard and agreed time points to provide good evidence on which services can progress quality improvement.

### 5.3. Ongoing support to services

IFs work with each service throughout their membership to ensure ongoing effective data collection and identification of quality improvement opportunities. This includes review of workflows and processes to ensure the collection of patient-reported outcome measures occurs at the correct time, and activities to increase user capability in respect of the epiCentre application.

IFs work directly with service staff to review their submitted data and identify records for revision/correction. Identification of data quality issues has seen the quality of service submitted data increase over time. Identification of data quality issues provides an opportunity for services to implement a quality approach to the data submission process. Over time, the quality of service submitted data increases. Obtaining sufficient outcome data is also important for the reliability of outcomes reporting and a necessary component for services to achieve a benchmark result. Benchmark results enable services to compare themselves to the aggregated data for all services and provide context for improvement of clinical practice. These outcomes provide an excellent starting point to identify clinical and service domains where results may be less than expected, prompting either review of workflows and processes or requests to ePPOC for quality improvement support and advice.

Report review meetings are offered to all participating pain services after they receive their six-monthly report. To assist, ePPOC provides a report review template to elicit information from the staff within each service. This prompts staff to reflect on their strengths and areas for improvement, concluding with an identified quality improvement goal. Offering routine service-specific support has been particularly important since the COVID pandemic, which forced changes to the way pain services delivered services, including a significant increase in online delivery of healthcare, leading to changes to group program content and service timeframes. The ongoing collection of data twice per year has allowed services to compare the outcomes of interventions delivered remotely with those delivered face to face.

ePPOC have found that services who regularly schedule a report review meeting are more likely to better understand their report data and use this to drive quality improvement. IFs also provide a valuable link between pain services for the purposes of sharing knowledge within the wider pain sector. Knowledge sharing and networking is also facilitated by ePPOC through the annual Australasian adult and pediatric benchmarking workshops, which are open to all member services. These workshops provide a further opportunity for services to gain insight into the wider sector and the outcomes being generated across Australia and New Zealand, promoting a community of practice.

### 5.4. Quality improvement as the “Engine Room” of outcomes collaboration

As discussed above, the IF role serves an important liaison role, facilitating bidirectional communication between the participating pain services and the wider ePPOC team, which is comprised of statisticians, a director, and administrative and information technology support staff. ePPOC is first and foremost a data collection designed to be collected and used at the point of care. Pain services are well-positioned to advise on the utility of ePPOC in their daily practice. As the designated point of first contact for services, the IFs receive valuable feedback from services on their experience with the program and can communicate this to the broader ePPOC team.

The IF role is now an established and essential component to the success of the ePPOC program, playing an important role in education, support, maintaining continued engagement, and facilitating communication between the member services and the broader ePPOC team. This role continues to evolve in line with changing internal and external environments. Historically, much of the focus has been on upskilling member services to maximize capability. As more services become proficient in their use of ePPOC, the IF role will need to pivot and provide adjusted offerings to accommodate the different needs of these services, which are likely to involve a deeper understanding of their data set, and individualized quality improvement initiatives.

## 6. Research outputs

### 6.1. External and internal research

Over the decade since ePPOC's establishment, there has been increasing interest in use of the ePPOC datasets for research purposes. ePPOC's binational dataset has provided a unique opportunity for studies to look at results across a large cohort of services from two countries, with a wide range of treatment modalities. The ability to study outcomes across a large dataset provides opportunities for customization of care and improvement of quality (13). The list of published articles using the ePPOC dataset can be found on the ePPOC website (9), and research to date illustrates the diversity of topics the dataset can engender. This has included opioid cessation impacts on pain and function, outcomes inequities by geographical location and patterns of patient outcomes in specialist pain management units. As occurs with the work undertaken by IFs with participating pain services, there is a quality improvement component of research, with the external applicants providing valuable insights into ways the ePPOC dataset can be itself improved.

ePPOC also publishes research findings as an information series on the ePPOC website (13), which are subject to the same governance and approval processes as external research using the ePPOC dataset. The publication of the information series helps to disseminate ePPOC research findings to pain services, who in turn can utilize the findings to improve pain management outcomes.

### 6.2. Future research

As discussed above, research is a core function of ePPOC and requests for access to the ePPOC dataset continue to increase. Planning is underway for the third iteration of the ePPOC dataset to reflect changing pain service and research needs in both the adult and pediatric spheres. A focus of research for ePPOC staff in the short to medium term will include investigation of the efficacy of a shortened dataset for some clinical applications, the impact of telehealth on pain service outcomes and more detailed study on waiting times and outcomes. The ePPOC research strategy will continue to be informed by member services, with guidance from ePPOC's governance committee (CMAC).

## 7. Discussion

The first ten years of ePPOC's existence has seen its dataset used to undertake research and quality improvement initiatives that are improving understanding of chronic pain and effective pain management. This ongoing maturity of approach has a substantial impact, both in respect of human factors (improved understanding of chronic pain, the lived experience of pain amongst diverse populations and geographies) and in respect of system factors (increased knowledge of treatment modalities and enhancing service approaches to improve outcomes). This evolution is continuous, with a strategic review of ePPOC identifying the need for further improvements in information technology and customization of the dataset and reports for different service needs.

Since the report on the establishment of ePPOC (1), the number of participating services has doubled to more than 100, further strengthening the dataset and the potential impact of quality improvement initiatives.

## 8. Conclusion

After a decade of operation, ePPOC has matured to be a well-regarded outcomes collaboration/registry. Like the wider chronic pain sector, there is constant change and evolution. The provision of robust outcomes data framed within a quality improvement context, allows pain services to have reliable guidance on their impact and the potential for further impact as quality improves further. If sustainability of funding and continued close liaison with the chronic pain sector occurs, there remains the opportunity for the ePPOC dataset to be an ongoing source of both outcomes and registry information that can inform practice at the local, funder and governmental policy levels.

## Data Availability

The data analyzed in this study is subject to the following licenses/restrictions: Access to the dataset are bound by the Australian Health Services Research Institute Data Access policy, requiring application to the corresponding author. Requests to access these datasets should be directed to David Holloway, eppoc-uow@uow.edu.au.
